# Changes in the perceived quality of primary care in Shanghai and Shenzhen, China: a difference-in-difference analysis

**DOI:** 10.2471/BLT.14.139527

**Published:** 2015-04-16

**Authors:** Xiaolin Wei, Haitao Li, Nan Yang, Samuel YS Wong, Marc CS Chong, Leiyu Shi, Martin CS Wong, Jianguang Xu, Dan Zhang, Jinling Tang, Donald KT Li, Qingyue Meng, Sian M Griffiths

**Affiliations:** a 2/F School of Public Health, Prince of Wales Hospital, Shatin, New Territories, Hong Kong Special Administrative Region (SAR), China.; b School of Public Health, Johns Hopkins University, Baltimore, United States of America.; c Shanghai Municipal Commission of Health and Family Planning, Shanghai, China.; d Shenzhen Municipal Commission of Health and Family Planning, Shenzhen, China.; e Bauhinia Foundation Research Centre, Hong Kong SAR, China.; f China Centre for Health Development Studies, Peking University, China.

## Abstract

**Objective:**

To assess changes in the quality of primary care in two megacities following the introduction of health system reforms in China.

**Methods:**

We conducted multistage stratified random face-to-face surveys of patients visiting community health centres in Shanghai in 2011 and 2013, and Shenzhen in 2012 and 2013. Quality of primary care was measured using an assessment tool. Difference-in-difference analyses based on multiple linear regressions were used to compare the changes over time, after controlling for potential confounders.

**Findings:**

Most (2721) of the 3214 participants used a community health centre as their regular source of care and were included in our analyses. The mean total scores for quality of primary care were similar for Shanghai and Shenzhen at baseline. In Shenzhen, the mean total scores for all participants and those on low incomes had worsened by 0.922 (95% CI: 0.629 to 1.215) and 1.203 (95% CI: 0.397 to 2.009), respectively. In Shanghai, however, there were improvements in the mean total scores which included increases in the scores for first-contact utilization, continuity, coordination of information and comprehensiveness.

**Conclusion:**

The quality of primary care improved in Shanghai but not in Shenzhen. This may be because, in Shanghai, beneficial long-term relationships between patients and general practitioners were supported by capitation payments and the provision of services tailored to the local health priorities.

## Résumé

**Objectif:**

Évaluer les changements dans la qualité des soins de santé primaires, dans deux mégapoles chinoises, après l'introduction de réformes dans le système de santé en Chine.

**Méthodes:**

Nous avons réalisé des enquêtes en face à face aléatoires stratifiées à plusieurs degrés auprès des patients des centres de santé communautaires de Shanghai en 2011 et 2013 et de Shenzhen en 2012 et 2013. La qualité des soins de santé primaires a été mesurée à l'aide d'un outil d'évaluation. Des analyses de l'écart des différences basées sur des régressions linéaires multiples ont été réalisées afin de comparer les changements survenus au fil du temps, après contrôle des facteurs de confusion potentiels.

**Résultats:**

Sur les 3 214 participants, la majorité (2 721 personnes) a indiqué fréquenter régulièrement le centre de santé communautaire et a été incluse dans notre analyse. Au départ, les scores totaux moyens attribués à la qualité des soins primaires ont été similaires à Shanghai et à Shenzhen. À Shenzhen, les scores totaux moyens attribués par tous les participants et par les participants à faible revenu ont respectivement baissé de 0,922 (IC 95 % : de 0,629 à 1,215) et de 1,203 (IC 95% : de 0,397 à 2,009). À Shanghai, en revanche, les scores totaux moyens ont progressé, notamment avec l'amélioration des scores associés aux critères « utilisation de premier recours », « continuité », « coordination de l'information » et « exhaustivité ».

**Conclusion:**

La qualité des soins primaires s'est améliorée à Shanghai mais pas à Shenzhen. Cela est peut-être lié au fait qu'à Shanghai, les relations bénéfiques de longue date instaurées entre les patients et les généralistes ont été soutenues par des paiements par capitation et par la fourniture de services adaptés aux priorités de santé locales.

## Resumen

**Objetivo:**

Evaluar los cambios en la calidad de la atención primaria en dos megaciudades tras la introducción de las reformas del sistema sanitario en China.

**Métodos:**

Se llevaron a cabo encuestas cara a cara aleatorias y estratificadas en varias etapas a pacientes que visitaron los centros sanitarios comunitarios de Shanghai en 2011 y 2013 y de Shenzhen en 2012 y 2013. La calidad de la atención primaria se midió utilizando una herramienta de evaluación. Los análisis de diferencias en diferencias basados en regresiones lineales múltiples se utilizaron para comparar los cambios a lo largo del tiempo tras haber controlado los posibles factores de confusión.

**Resultados:**

La mayoría (2.721) de los 3.214 participantes utilizaban un centro sanitario comunitario como su fuente regular de atención primaria y fueron incluidos en nuestros análisis. Las puntuaciones medias totales de la calidad de la atención primaria eran similares para Shanghai y Shenzhen al inicio del estudio. En Shenzhen, las puntuaciones medias totales de todos los participantes y de aquellos con bajos ingresos había empeorado en un 0,922 (IC del 95%: de 0,629 a 1,215) y un 1,203 (IC del 95: de 0,397 a 2,009), respectivamente. Sin embargo, en Shanghai hubo mejoras en las puntuaciones medias totales, incluidos incrementos en las puntuaciones de la utilización de primer contacto, la continuidad, la coordinación de la información y la exhaustividad.

**Conclusión:**

La calidad de la atención primaria mejoró en Shanghai pero no en Shenzhen. Esto se puede deber a que en Shanghai, las relaciones beneficiosas a largo plazo entre los pacientes y los médicos generales estaban respaldadas por pagos de capitación y la prestación de servicios adaptados a las prioridades sanitarias locales.

## ملخص

الغرض تقييم التغيرات في جودة الرعاية الصحية الأولية في اثنتين من المدن الكبرى في أعقاب إدخال إصلاحات على النظام الصحي في الصين.

الطريقة أجرينا مسوحات عشوائية متطبِّقة متعددة المراحل وجهًا لوجه للمرضى الذين يترددون على المراكز الصحية المجتمعية في شانغهاي في عاميّ 2011 و2013، وشنجن في عاميّ 2012 و2013. وقد تم قياس جودة الرعاية الصحية الأولية باستخدام أداة للتقييم. تم استخدام أسلوب الاختلافات في الفارق المستند إلى التحوفات الخطية المتعددة لمقارنة التغيرات بمرور الوقت، بعد ضبط المتغيرات المحيّرة المحتملة.

النتائج استخدم معظم المشاركين البالغ عددهم (2721) من أصل 3214 مشاركًا المراكز الصحية المجتمعية كمصدر منتظم للرعاية وتم تضمينهم في التحليل الذي أجريناه. وكانت الأحراز الكلية الوسطية لجودة الرعاية الصحية الأولية مشابهة لشانغهاي وشنجن عند خط الأساس. في شنجن، ساءت متوسط الأحراز الكلية لجميع المشاركين والأشخاص ذوي الدخل المنخفض بنسبة تبلغ 0.922 (بنسبة أرجحية مقدارها 95%: 0.629 إلى 1.215) و1.203 (بنسبة أرجحية مقدارها 95%: 0.397 إلى 2.009)، على التوالي. أما في شانغهاي، فقد كانت هناك تحسينات في متوسط الأحراز الكلية والتي تضمنت زيادات في أحراز الانتفاع للاتصال الأول، والاستمرارية، وتنسيق المعلومات وشموليتها.

الاستنتاج لقد تحسنت جودة الرعاية الصحية الأولية في شانغهاي لكنها لم تتحسن في شنجن. ويمكن تفسير ذلك بأنه في شنغهاي، تم دعم علاقات مفيدة طويلة الأمد بين المرضى والممارسين العموميين من خلال دفع الأجرة على الفرد وتوفير خدمات توضع تبعًا لأولويات الصحة المحلية.

## 摘要

**目的:**

旨在评估初级医疗质量在中国实行卫生系统改革的两个特大城市中发生的变化。

**方法:**

我们针对 2011 年和 2013 年在上海以及 2012 年和 2013 年在深圳就诊于社区卫生中心的患者开展了一项多阶段、分层、随机式的面对面调查。通过评估工具对初级医疗的质量进行了衡量。基于多元线性回归的双重差分法分析用于对比在控制了潜在的混杂因素后,一段时间内发生的变化。

**结果:**

3214 名参与者中的大多数人 (2721) 都将社区卫生中心作为自己常规就诊的医疗机构,这些都包括在我们的分析中。上海和深圳的初级医疗质量的总均分在基准值上很相似。在深圳,所有参与者和低收入人群的总均分已分别降低了 0.922(95% CI:0.629 至 1.215)和 1.203(95% CI:0.397 至 2.009)。然而在上海,总均分却有所提高,这种情况包括在第一线就诊渠道利用率、持续性、信息协调性和全面性方面的分值提高。

**结论:**

上海的初级医疗质量有所提高,而深圳并非如此。这可能是因为,在上海实行按人头付费并依据当地卫生工作重点提供服务,促使患者与全科医生之间保持长期有益的关系。

## Резюме

**Цель:**

Оценка изменения качества первичной медицинской помощи в двух мегаполисах после проведения реформ системы здравоохранения в Китае.

**Методы:**

Был проведен многоступенчатый стратифицированный индивидуальный опрос выбранных случайным образом пациентов, посещающих общественные медицинские центры в Шанхае (2011 и 2013 гг.) и Шэньчжэне (2012 и 2013 гг.). Качество первичной медицинской помощи определялось с помощью специального инструмента оценки. Для сравнения изменений с течением времени после отсева искажающих факторов использовался метод «разность разностей» на основе множественной линейной регрессии.

**Результаты:**

Большинство участников (2721 из 3214) регулярно пользуются услугами общественных медицинских центров для получения медицинской помощи, и они были включены в наш анализ. Средняя общая оценка качества первичной медицинской помощи в начале исследования в Шанхае и Шэньчжэне была одинаковой. В Шэньчжэне средняя общая оценка для всех участников и лиц с низкими доходами ухудшилась на 0,922 (95% ДИ: с 0,629 до 1,215) и 1,203 (95% ДИ: с 0,397 до 2,009) соответственно. С другой стороны, в Шанхае отмечались некоторые улучшения средней общей оценки: были получены более высокие оценки в отношении качества обслуживания при первом обращении, непрерывности лечения, организации информационного процесса и комплексности лечения.

**Вывод:**

Качество первичной медицинской помощи в Шанхае улучшилось, однако в Шэньчжэне этого не произошло. Причиной может быть то, что в Шанхае выгодные долгосрочные отношения между пациентами и врачами общей практики поддерживались за счет подушных платежей и предоставления услуг с учетом местных приоритетов в области здравоохранения.

## Introduction

China’s health system, which serves a population of 1.3 billion, is the world’s largest. Primary care facilities consisting of urban community health centres, rural township hospitals and village clinics provide clinical care, disease prevention activities, health promotion, rehabilitation and family planning. Since the 1980s, market reform in China has brought rapid economic development and privatization of health care. Government funding of health care has fallen, the scheme for rural health insurance has been dismantled and out-of-pocket payments have increased. At the same time, health inequity has increased and hospitals have become the main providers of acute care.^1^

In an attempt to reverse the deterioration in primary care, the Chinese government launched a systemic health reform in 2009. The aim of the reform was the provision of affordable and equitable health care for all by 2020. As a result of the subsequent substantial infusion of public funding, primary care now occupies a central role in the rebuilding of a public-oriented health system in China. Since 2009, health insurance coverage has expanded rapidly, with the majority of citizens now covered,^2^ and patient access to primary care facilities has improved.^3^ In 2011, the national government issued policies that focused on enhancing the public financing of primary care facilities, strengthening the training of general practitioners and a service model based on general practitioner teams. The teams – which consist of a general practitioner and a nurse and, sometimes, a public health specialist – provide clinical and preventive care to enrolled residents.^4^

Implementation of the national policies began in Shanghai in 2011 and was expanded to cover Shenzhen in 2012. It is hoped that experience gained from the two megacities will facilitate improvements in the delivery of primary care in the rest of China (a megacity is defined as a metropolitan area with a total population exceeding 10 million people). Community health centres in the two megacities provide similar clinical and public health care according to the national guidelines.^5^ The clinical services include treatment of common diseases in internal medicine, general surgery, paediatrics, gynaecology, obstetrics and traditional Chinese medical care. The public health services provided in the community health centres are entirely funded by the government and include programmes for the management of diabetes and hypertension, health education, immunization, maternal and child health care, mental health care and communicable disease prevention and reporting.^6^ Internal migrants make up 80% of Shenzhen’s residents but only 30% of Shanghai’s; less than 5% of Shenzhen’s residents – but 27% of Shanghai’s – are aged at least 60 years.^7^

A community health centre in Shanghai covers 50 000–100 000 residents and usually contains between five and eight general practitioner teams.^8^ In Shenzhen, a community health centre covers 30 000–50 000 residents and contains two or three general practitioner teams.^9^ The community health centres in Shanghai are fully government-funded and independent of public hospitals. Any revenues they generate through service provision go directly to the government. Shanghai’s general practitioners are also fully publicly funded and most of them received five years of medical training as undergraduates.^10^ In contrast, Shenzhen’s community health centres are subsidiaries of public hospitals that are allowed to make profits to cover most of their own costs. Public health services in Shenzhen are funded by the municipal government and its general practitioners are hospital employees who have to earn their own salaries through general practice.^10^ Most of Shenzhen’s general practitioners received only three years of medical training.^10^

Shanghai promoted the service model based on general practitioner teams by introducing capitation payments for general practitioners and extended benefit packages for residents who enrolled on the teams.^11^ Although Shenzhen implemented the national policy on the same model, it made no operational changes specifically to promote the model.^12^ Both megacities promoted the use of community health centres by introducing preferential prices and encouraging referrals between the centres and hospitals.

We decided to investigate if the health reforms improved the patient-perceived quality of primary care in the two megacities.

## Methods

### Participants

We measured quality in terms of eight different attributes of primary care, using an internationally recognized assessment tool.^13^^–^^20^Questionnaire-based face-to-face surveys were conducted in two rounds. The first round of the survey was conducted – to coincide with the local introduction of the national policies on primary care – in November 2011 in Shanghai and in June 2012 in Shenzhen. The second round was conducted in June 2013 in Shanghai and in August 2013 in Shenzhen. Based on an estimated difference in the mean total primary care score between the two survey rounds of 0.8, with a standard deviation of 5,^18^ a 95% confidence interval (CI) and a power of 80% – we estimated the minimum sample size in each megacity to be 614. After assuming that 90% of those invited to participate would participate and that 85% of participants would regard a community health centre as their usual source of care, we aimed to interview 800 individuals in each megacity in each round of the survey. After stratifying each megacity into four geographical areas, we used a computer-generated random sequence to select one study district in each of the areas. Then – again using a computer-generated random sequence – we selected one community health centre in each study district. In each megacity, the two rounds of surveys were conducted in the same four community health centres but potential participants were randomly approached in each round. In each surveyed community health centre – until the number of participants from the centre reached 200 – every fifth care user aged at least 18 years was invited to participate in the survey. Each participant was fully informed of the purpose of the study and provided written informed consent before being interviewed.

### Evaluating quality

The primary care assessment tool was translated into Chinese and validated in a pilot study (available from corresponding author).^13^^,^^21^ This tool measures the patient-perceived quality of primary care in terms of eight attributes: first-contact utilization, first-contact accessibility, continuity of care, coordination of services, coordination of information, comprehensiveness of the available service, comprehensiveness of the provided service and a measure of how well the care was patient-focused (so-called patient-centredness). For each of these attributes, patients are asked how well they agree with a statement that their regular provider of primary care is excellent in terms of the attribute. Using a four-point Likert-type scale, each patient’s level of agreement with each statement is scored from 1 (indicating that the patient definitely does not agree) to 4 (indicating that the patient definitely does agree). The maximum total score is 32. In our study, we recorded scores and the demographic and socioeconomic characteristics of each participant, their self-reported health status and their diagnosed chronic disease, if any. We classified employment status simply into those who were employed and those who were unemployed. The latter category included participants who had retired. Any household with a monthly income below the value taken as the poverty line in China in 2011 – i.e. 484 United States dollars (US$) – was considered to have a low income whereas any household with a monthly income above the median value for the two megacities in 2011 – i.e. US$ 1613 – was considered to have a high income.^22^^,^^23^ Households with an income between low and high-income level were considered middle income-level.

### Analysis

We used multiple linear regression models to compare the scores for the quality of primary care recorded in the two megacities at baseline and in each megacity during the two survey rounds. We adjusted for several potential confounders: sex, age, marital status, migrant status, educational level, employment status, household income, health insurance status, number of visits to the community health centre in the previous 12 months, number of years since the patient’s first visit to the community health centre, self-reported health status and presence of diagnosed chronic disease. To compare the effect of policy changes in the two megacities over time, we applied difference-in-difference analysis using equation (1):
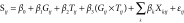
(1)where *S_ij_* is the primary care score given by the *i*th participant in the *j*th megacity, *G_ij_* indicates the megacity, *T_ij_* indicates the survey round, the interaction term *G_ij_* × *T_ij_* indicates the difference in the between-round changes in each megacity, *X_kij_* represents the potential confounder *k* and *β*_0_ and *ε_ij_* represent the intercept and error term in the model, respectively. The difference in the between-round changes in the quality scores for each megacity yielded an estimate of *β*_3_. We ran three separate regression models – one that included all of the participants, one restricted to those participants who had been diagnosed as having a chronic disease, and one restricted to the participants who came from poor households. All analyses were conducted using SPSS version 19.0 (SPSS Inc., Chicago, United States of America).

### Ethical approval

Ethical approval was obtained from the joint Chinese University of Hong Kong and New Territories East Cluster Clinical Research ethics committees (Hong Kong Special Administrative Region, China; reference CRE-2012.441).

## Results

### Participant characteristics

In Shanghai, 811 (94%) of the 860 patients invited to participate in the first survey round and 795 (92%) of the 861 invited to participate in the second round completed the questionnaire. The corresponding values for Shenzhen were 806 (85%) of 947 and 802 (84%) of 954, respectively. Of the patients who completed the questionnaire in the first and second rounds, 725 (89%) and 741 (93%) of those in Shanghai and 640 (79%) and 615 (77%) of those in Shenzhen, respectively, said that they regarded the community health centre in which they were recruited as their usual source of care and were therefore included in the subsequent analyses (Table 1). In both megacities, most participants were female, married and educated to below college level, belonged to a household that was neither poor nor rich and had health insurance (Table 1). Most of the participants in Shanghai were non-migrants older than 59 years who did not have a job. Most had visited the community health centre where they were recruited more than 13 times in the previous 12 months, had first visited that centre more than five years previously and had a chronic disease when they participated in a survey round. In Shenzhen, in contrast, most of the participants were migrants in employment and younger than 45 years. Compared with those in Shanghai, the participants in Shenzhen were more likely to have a low educational level, come from poor households and lack health insurance but were less likely to have a diagnosed chronic disease and tended to have visited the community health centre where they were recruited less frequently in the previous 12 months (Table 1).

**Table 1:** Characteristics of the participants in the perceived quality-of-care surveys, Shanghai and Shenzhen, China, 2011–2013

Characteristic	No. of participants in Shanghai (%)				No. of participants in Shenzhen (%)		
	First round (*n* = 725)	Second round^a^ (*n* = 741)	Both rounds^b^ (*n* = 1466)		First round (*n* = 640)	Second round^a^ (*n* = 615)	Both rounds^b^ (*n* = 1255)
**Sex**							
Female	497 (68.6)	506 (68.3)	1003 (68.4)		430 (67.2)	402 (65.4)	832 (66.3)
Male	228 (31.4)	235 (31.7)	463 (31.6)		210 (32.8)	213 (34.6)	423 (33.7)
**Age (years)**							
≤ 44	39 (5.4)	42 (5.7)**	81 (5.5)		482 (75.3)	484 (78.7)	966 (77.0)***
45–59	214 (29.5)	160 (21.6)	374 (25.5)		98 (15.3)	69 (11.2)	167 (13.3)
≥ 60	472 (65.1)	539 (72.7)	1011 (69.0)		60 (9.4)	62 (10.1)	122 (9.7)
**Marital status**							
Single	102 (14.1)	85 (11.5)	187 (12.8)		148 (23.1)	117 (19.0)	265 (21.1)***
Married	623 (85.9)	656 (88.5)	1279 (87.2)		492 (76.9)	498 (81.0)	990 (78.9)
**Migrant status**							
Non-migrant	674 (93.0)	702 (94.7)	1376 (93.9)		114 (17.8)	83 (13.5)*	197 (15.7)***
Migrant	51 (7.0)	39 (5.3)	90 (6.1)		526 (82.2)	532 (86.5)	1058 (84.3)
**Education**							
Middle school or below	307 (42.3)	289 (39.0)**	596 (40.7)		292 (45.6)	277 (45.0)	569 (45.3)*
High school or equivalent	267 (36.8)	241 (32.5)	508 (34.7)		202 (31.6)	218 (35.4)	420 (33.5)
College and above	151 (20.8)	211 (28.5)	362 (24.7)		146 (22.8)	120 (19.5)	266 (21.2)
**Employment**							
Employed	94 (13.0)	94 (12.7)	188 (12.8)		488 (76.3)	434 (70.6)*	922 (73.5)***
Unemployed	631 (87.0)	647 (87.3)	1278 (87.2)		152 (23.8)	181 (29.4)	333 (26.5)
**Household income category**							
Low	111 (15.3)	79 (10.7)***	190 (13.0)		94 (14.7)	105 (17.1)	199 (15.9)***
Middle	542 (74.8)	516 (69.6)	1058 (72.2)		404 (63.1)	385 (62.6)	789 (62.9)
High	72 (9.9)	146 (19.7)	218 (14.9)		142 (22.2)	125 (20.3)	267 (21.3)
**Health insurance**							
Insured	692 (95.4)	714 (96.4)	1406 (95.9)		494 (77.2)	414 (67.3)***	908 (72.4)***
Uninsured	33 (4.6)	27 (3.6)	60 (4.1)		146 (22.8)	201 (32.7)	347 (27.6)
**CHC visits in previous 12 months**							
≤ 4	80 (11.0)	69 (9.3)	149 (10.2)		385 (60.2)	425 (69.1)**	810 (64.5)***
5–12	141 (19.4)	164 (22.1)	305 (20.8)		207 (32.3)	144 (23.4)	351 (28.0)
≥ 13	504 (69.5)	508 (68.6)	1012 (69.0)		48 (7.5)	46 (7.5)	94 (7.5)
**Time since first visit to CHC (years)**							
≤ 2	149 (20.6)	99 (13.4)***	248 (16.9)		356 (55.6)	377 (61.3)*	733 (58.4)***
3–4	94 (13.0)	79 (10.7)	173 (11.8)		147 (23.0)	116 (18.9)	263 (21.0)
≥ 5	482 (66.5)	563 (76.0)	1045 (71.3)		137 (21.4)	122 (19.8)	259 (20.6)
**Health status**							
Good	174 (24.0)	207 (27.9)*	381 (26.0)		326 (50.9)	343 (55.8)	669 (53.3)***
Fair	449 (61.9)	458 (61.8)	907 (61.9)		275 (43.0)	237 (38.5)	512 (40.8)
Poor	102 (14.1)	76 (10.3)	178 (12.1)		39 (6.1)	35 (5.7)	74 (5.9)
**Diagnosed chronic disease**^c^							
Present	600 (82.8)	578 (78.0)*	1178 (80.4)		129 (20.2)	108 (17.6)	237 (18.9)***
Absent	125 (17.2)	163 (22.0)	288 (19.6)		511 (79.8)	507 (82.4)	1018 (81.1)

CHC: community health centre. * *P* < 0.05; ** *P* < 0.01; *** *P* < 0.001.

^a^ The indicated *P*-values came from χ^2^ tests in which, for each megacity, the values recorded in the first and second rounds were compared.

^b^ The indicated *P*-values came from χ^2^ tests in which the values recorded across both rounds in Shanghai were compared with the corresponding values recorded in Shenzhen.

^c^ Hypertension, diabetes, cardiovascular, chronic respiratory, pulmonary, liver, thyroid or skeleto–muscular diseases, gastrointestinal disorders, mental illness and/or disability.

### Quality scores

#### All participants

The mean total quality scores recorded in the first survey round, after adjusting for potential confounders, were similar in the two megacities: 23.18 (standard deviation, SD: 2.52) in Shanghai and 22.77 (SD: 2.75) in Shenzhen (Table 2). In Shanghai, mean total scores increased by 0.712 (95% CI: 0.457 to 0.967) in the second survey round, the scores for first contact, continuity, coordination of information and comprehensiveness and the second-round total score were significantly higher whereas the second-round scores for coordination of services and patient-focused care were significantly lower (*P* < 0.05 for each comparison). In Shenzhen, however, the mean total scores had fallen by 0.922 (95% CI: 0.629 to 1.215) in the second survey round. All except those for first-contact accessibility were lower than the corresponding first-round values. The results of the difference-in-difference analysis indicated that Shanghai experienced much greater improvements in the quality of primary care between the two survey rounds (1.651, 95% CI: 1.266 to 2.037) – at least in terms of first-contact utilization, continuity, coordination of information and comprehensiveness – than Shenzhen (Table 2).

**Table 2:** Quality scores given, for primary care, by all of the participants in the perceived quality-of-care surveys, Shanghai and Shenzhen, China, 2011–2013

Attribute	Shanghai					Shenzhen			Difference in differences (95% CI)^a^
	Score (SD)			Difference^b^ score (95% CI)		Score (SD)		Difference^b^ score (95% CI)	
	First round (*n* = 725)		Second round (*n* = 741)			First round^c^ (*n* = 640)	Second round (*n* = 615)		
**First contact**									
Utilization	2.54 (0.58)		2.69 (0.57)	0.121 (0.066 to 0.177)		2.58 (0.50)**	2.47 (0.53)	−0.117 (−0.172 to −0.063)	0.255 (0.176 to 0.333)
Accessibility		2.45 (0.30)	2.53 (0.35)	0.077 (0.044 to 0.111)		2.57 (0.36)*	2.68 (0.37)	0.108 (0.067 to 0.148)	−0.030 (−0.082 to 0.022)
**Continuity of care**		3.25 (0.44)	3.37 (0.45)	0.121 (0.076 to 0.167)		3.20 (0.47)***	3.07 (0.44)	−0.117 (−0.167 to −0.068)	0.233 (0.166 to 0.300)
**Coordination**									
Services		2.56 (0.54)	2.49 (0.64)	−0.080 (−0.142 to −0.018)		2.42 (0.61)*	2.25 (0.63)	−0.148 (−0.218 to −0.079)	0.083 (−0.009 to 0.175)
Information		3.64 (0.48)	3.82 (0.40)	0.175 (0.129 to 0.221)		3.23 (0.69)***	3.16 (0.69)	−0.068 (−0.146 to 0.009)	0.233 (0.147 to 0.320)
**Comprehensiveness**									
Service availability		3.42 (0.40)	3.55 (0.31)	0.113 (0.077 to 0.150)		3.24 (0.44)	3.02 (0.51)	−0.208 (−0.261 to −0.155)	0.325 (0.262 to 0.388)
Service provided		2.43 (0.60)	2.71 (0.59)	0.275 (0.213 to 0.337)		2.48 (0.67)***	2.30 (0.59)	−0.184 (−0.253 to −0.116)	0.454 (0.362 to 0.546)
**Patient-focused care**		2.89 (0.84)	2.81 (0.83)	−0.091 (−0.178 to −0.004)		3.05 (0.83)	2.87 (0.76)	−0.186 (−0.275 to −0.097)	0.098 (−0.027 to 0.222)
**All**		23.18 (2.52)	23.97 (2.43)	0.712 (0.457 to 0.967)		22.77 (2.75)	21.81 (2.58)	−0.922 (−1.215 to −0.629)	1.651 (1.266 to 2.037)

CI: confidence interval; SD: standard deviation. * *P* < 0.05; ** *P* < 0.01; *** *P* < 0.001.

^a^ As estimated in multiple linear regression models after adjusting for potential confounders, with the Shenzhen scores used as the reference.

^b^ The trend in the score between the first and second rounds, as seen in multiple linear regression models after adjusting for potential confounders.

^c^ The indicated *P*-values came from multiple linear regression models in which, after adjusting for potential confounders, the values recorded in the first round in Shanghai were compared with those recorded in the first round in Shenzhen.

#### Participants with chronic diseases

At least one diagnosed chronic disease was reported by each of 1178 participants in Shanghai and 237 participants in Shenzhen (Table 3). In the first survey round, there was little difference in the quality scores given by participants with chronic disease in Shanghai (23.18; SD: 2.57) and Shenzhen (23.10; SD: 2.89). In the second round, the score had increased to 23.96 (SD: 2.38) in Shanghai. The participants with chronic disease gave scores for all attributes – except coordination of services – that were significantly higher than the corresponding first-round values. In Shenzhen, the score had fallen to 22.54 (SD: 2.74) and the only score reported in the second round that was significantly different to the corresponding first-round value was the score for comprehensiveness of service availability – which was significantly lower. The results of the difference-in-difference analysis indicated that Shanghai’s participants with diagnosed chronic disease experienced much greater improvements in the quality of primary care between the two survey rounds – at least in terms of first-contact utilization, continuity, coordination of information and comprehensiveness – than their counterparts in Shenzhen (Table 3).

**Table 3:** Quality scores given, for primary care, by the participants with diagnosed chronic disease who were included in the perceived quality-of-care surveys, Shanghai and Shenzhen, China, 2011–2013

Attribute	Shanghai				Shenzhen			Difference in differences (95% CI)^a^
	Score (SD)		Difference^b^ score (95% CI)		Score (SD)		Difference^b^ score (95% CI)	
	First round (*n* = 600)	Second round (*n* = 578)			First round^c^ (*n* = 129)	Second round (*n* = 108)		
**First contact**								
Utilization	2.57 (0.58)	2.74 (0.55)	0.140 (0.078 to 0.202)		2.55 (0.49)*	2.54 (0.55)	−0.067 (−0.190 to 0.055)	0.174 (0.026 to 0.323)
Accessibility	2.44 (0.31)	2.51 (0.34)	0.066 (0.029 to 0.104)		2.54 (0.36)	2.66 (0.38)	0.095 (0.000 to 0.193)	−0.044 (−0.138 to 0.050)
**Continuity of care**	3.26 (0.44)	3.39 (0.44)	0.131 (0.081 to 0.182)		3.27 (0.53)**	3.17 (0.43)	−0.079 (−0.204 to 0.046)	0.204 (0.080 to 0.329)
**Coordination**								
Services	2.56 (0.54)	2.47 (0.64)	−0.111 (−0.180 to −0.042)		2.46 (0.60)	2.33 (0.62)	−0.122 (−0.290 to 0.046)	−0.001 (−0.170 to 0.170)
Information	3.63 (0.48)	3.83 (0.38)	0.184 (0.133 to 0.235)		3.26 (0.70)***	3.16 (0.71)	−0.131 (−0.324 to 0.061)	0.275 (0.136 to 0.413)
**Comprehensiveness**								
Service availability	3.42 (0.40)	3.54 (0.30)	0.103 (0.062 to 0.143)		3.28 (0.44)	3.12 (0.48)	−0.125 (−0.247 to −0.002)	0.216 (0.111 to 0.320)
Service provided	2.45 (0.60)	2.71 (0.58)	0.257 (0.188 to 0.326)		2.68 (0.63)***	2.57 (0.56)	−0.123 (−0.277 to 0.031)	0.347 (0.181 to 0.513)
**Patient-focused care**	2.86 (0.85)	2.78 (0.84)	0.014 (−0.044 to 0.071)		3.04 (0.86)	2.98 (0.83)	0.018 (−0.112 to 0.149)	−0.083 (−0.324 to 0.158)
**All**	23.18 (2.57)	23.96 (2.38)	0.672 (0.386 to 0.957)		23.10 (2.89)	22.54 (2.74)	−0.630 (−1.371 to 0.111)	1.089 (0.378 to 1.799)

CI: confidence interval; SD: standard deviation. * *P* < 0.05; ** *P* < 0.01; *** *P* < 0.001.

^a^ As estimated in multiple linear regression models after adjusting for potential confounders, with the Shenzhen scores used as the reference.

^b^ The trend in the score between the first and second rounds, as seen in multiple linear regression models after adjusting for potential confounders.

^c^ The indicated *P*-values came from multiple linear regression models in which, after adjusting for potential confounders, the values recorded in the first round in Shanghai were compared with those recorded in the first round in Shenzhen.

#### Participants from poor households

Overall, 389 participants came from poor households (Table 4). In the first survey round, the participants from poor households in Shanghai and Shenzhen reported similar quality scores (23.12; SD: 2.55 and 23.14; SD: 2.68, respectively). Between the first and second rounds, the quality of the primary care received by participants from poor households in Shanghai was slightly improved (score: 23.88 SD: 2.17). In Shenzhen, there was a more substantial reduction (score: 21.92; SD: 2.65; Table 4).

**Table 4:** Quality scores given, for primary care, by the participants from poor households who were included in the perceived quality-of-care surveys, Shanghai and Shenzhen, China, 2011–2013

Attribute	Shanghai				Shenzhen			Difference in differences (95% CI)^a^
	Score (SD)		Difference^b^ score (95% CI)		Score (SD)		Difference^b^ score (95% CI)	
	First round (*n* = 111)	Second round (*n* = 79)			First round^c^ (*n* = 94)	Second round (*n* = 105)		
**First contact**								
Utilization	2.68 (0.55)	2.75 (0.62)	0.044 (−0.122 to 0.210)		2.83 (0.51)*	2.80 (0.49)	0.011 (−0.132 to 0.153)	0.080 (−0.132 to 0.292)
Accessibility	2.44 (0.30)	2.51 (0.33)	0.046 (−0.053 to 0.144)		2.59 (0.36)	2.62 (0.38)	0.079 (−0.024 to 0.192)	−0.008 (−0.152 to 0.136)
**Continuity of care**	3.19 (0.48)	3.38 (0.47)	0.124 (−0.022 to 0.270)		3.23 (0.46)**	3.09 (0.43)	−0.153 (−0.286 to −0.020)	0.285 (0.098 to 0.472)
**Coordination**								
Services	2.57 (0.46)	2.51 (0.59)	−0.082 (−0.246 to 0.081)		2.41 (0.54)*	2.20 (0.59)	−0.231 (−0.400 to −0.063)	0.196 (−0.027 to 0.419)
Information	3.61 (0.55)	3.82 (0.39)	0.264 (0.107 to 0.421)		3.32 (0.71)	3.14 (0.74)	−0.219 (−0.448 to 0.009)	0.394 (0.129 to 0.659)
**Comprehensiveness**								
Service availability	3.38 (0.45)	3.50 (0.28)	0.064 (−0.053 to 0.181)		3.21 (0.46)	2.90 (0.53)	−0.255 (−0.404 to −0.105)	0.408 (0.218 to 0.598)
Service provided	2.47 (0.62)	2.68 (0.56)	0.143 (−0.041 to 0.327)		2.52 (0.63)***	2.39 (0.61)	−0.182 (−0.365 to 0.000)	0.385 (0.139 to 0.631)
**Patient-focused care**	2.78 (0.84)	2.74 (0.79)	−0.028 (−0.186 to 0.131)		3.04 (0.87)	2.78 (0.83)	−0.252 (−0.516 to 0.011)	0.287 (−0.064 to 0.638)
**All**	23.12 (2.55)	23.88 (2.17)	0.544 (−0.218 to 1.306)		23.14 (2.68)	21.92 (2.65)	−1.203 (−2.009 to −0.397)	2.027 (0.967 to 3.087)

CI: confidence interval; SD: standard deviation. * *P* < 0.05; ** *P* < 0.01; *** *P* < 0.001.

^a^ As estimated in multiple linear regression models after adjusting for potential confounders, with the Shenzhen scores used as the reference.

^b^ The trend in the score between the first and second rounds, as seen in multiple linear regression models after adjusting for potential confounders.

^c^ The indicated *P*-values came from multiple linear regression models in which, after adjusting for potential confounders, the values recorded in the first round in Shanghai were compared with those recorded in the first round in Shenzhen.

### Other observations

Income level, migrant status and employment status showed no significant association with the perceived quality of primary care (Table 5). However, participants who had visited their community health centre relatively often in the previous 12 months and those who had first visited their community health centre a relatively long time ago tended to report relatively high scores (Table 5).

**Table 5:** Factors associated with changes in mean total quality scores for primary care, Shanghai and Shenzhen, China, 2011–2013

Variable^a^	Difference in differences in scores (95% CI)^b^		
	All participants (*n* = 2721)	Participants with chronic diseases (*n* = 1415)	Participants from poor households (*n* = 390)
**Interaction of survey round and city**	1.651 (1.266 to 2.037)	1.089 (0.378 to 1.799)	2.027 (0.967 to 3.087)
**Survey round**			
Second	−0.951 (−1.233 to −0.668)	−0.407 (−1.053 to 0.240)	−1.359 (−2.105 to −0.613)
**Megacity**			
Shanghai	−0.402 (−0.814 to 0.010)	−0.691 (−1.328 to −0.054)	−0.581 (−1.765 to 0.603)
**Sex**			
Male	−0.224 (−0.434 to −0.014)	0.057 (−0.234 to 0.347)	−0.244 (−0.849 to 0.361)
**Age in years**			
45–59	0.090 (−0.267 to 0.448)	0.466 (−0.202 to 1.134)	−0.160 (−1.107 to 0.788)
≥ 60	0.352 (−0.051 to 0.754)	0.587 (−0.110 to 1.284)	0.380 (−0.622 to 1.382)
**Marital status**			
Married	0.263 (−0.009 to 0.536)	0.623 (0.197 to 1.049)	0.351 (−0.204 to 0.906)
**Migrant status**			
Migrant	0.256 (−0.087 to 0.598)	0.135 (−0.421 to 0.690)	−0.224 (−1.251 to 0.802)
**Education**			
High school and equivalent	−0.211 (−0.435 to 0.014)	−0.009 (−0.314 to 0.295)	−0.348 (−0.933 to 0.238)
College and above	−0.228 (−0.493 to 0.038)	−0.410 (−0.768 to −0.052)	−0.416 (−1.343 to 0.510)
**Employment**			
Unemployed	0.185 (−0.111 to 0.482)	0.019 (−0.473 to 0.511)	−0.217 (−0.996 to 0.563)
**Household income category**			
Middle	−0.127 (−0.415 to 0.160)	−0.183 (−0.601 to 0.234)	–
High	−0.006 (−0.367 to 0.355)	0.002 (−0.528 to 0.531)	–
**Health insurance**			
Uninsured	0.041 (−0.260 to 0.342)	0.417 (−0.167 to 1.002)	1.034 (0.268 to 1.800)
**CHC visits in previous 12 months**			
5–12	0.628 (0.359 to 0.897)	0.631 (0.153 to 1.109)	0.296 (−0.442 to 1.034)
≥ 13	1.203 (0.890 to 1.517)	1.288 (0.821 to 1.754)	0.718 (−0.185 to 1.621)
**Years since first visit to CHC**			
3–4	−0.185 (−0.477 to 0.108)	−0.150 (−0.624 to 0.323)	−0.297 (−1.125 to 0.532)
≥ 5	0.334 (0.078 to 0.591)	0.610 (0.229 to 0.990)	0.215 (−0.494 to 0.925)
**Health status**			
Fair	0.303 (−0.039 to 0.645)	0.170 (−0.217 to 0.557)	0.070 (−0.705 to 0.846)
Good	0.625 (0.259 to 0.990)	0.460 (0.008 to 0.912)	0.244 (−0.627 to 1.115)
**Diagnosed with chronic disease**	−0.065 (−0.342 to 0.212)	–	0.133 (−0.649 to 0.915)

CHC: community health centre; CI: confidence interval.

^a^ In the models, the first survey round, Shenzhen, female, younger than 45 years, unmarried, educated to no more than middle-school level, employed, low household income, with health insurance, fewer than five visits to the community health centre in the previous 12 months, having visited the CHC for less than three years, poor health status and absence of diagnosed chronic disease were used as reference categories.

^b^ Difference-in-difference models were calculated with Shenzhen used as reference and adjustments made for other potential confounders.

## Discussion

We assessed the quality of primary care in two megacities in China, following introduction of health reforms. Between 2011 and 2013, quality of primary care – as perceived by all participants, participants from poor households or participants with diagnosed chronic disease – significantly improved in Shanghai. In Shenzhen, however, the quality of primary care appears to have declined. Compared with the other participants, those who were rich, non-migrant and/or employed did not give markedly higher scores – perhaps because the community health centres in the megacities provide free public health services to all residents. In settings where primary care is dominated by private providers, the care received by the rich tends to be of higher quality than that received by the poor.^24^

Although we investigated changes in primary care quality in the context of the local implementation of national policies, the study design prevented us from demonstrating a causal inference between the policies and the changes that we observed. Our initial survey rounds were conducted shortly after – rather than before – the introduction of policy reform in Shanghai and Shenzhen and we may therefore have missed some of the changes triggered by the reform. Since we only investigated patients attending four community health centres in each of the megacities we studied, we should not assume that our findings apply to the whole population.

Despite these limitations, it is clear that changes in the quality of care in Shanghai differed markedly from those observed in Shenzhen. Residents in the catchment areas of community health centres in Shanghai are invited to sign a contract with one of the centre’s teams so that they can receive a package of clinical and preventive care – focused on diabetes and hypertension management, care of the elderly and timely referrals to specialists in large hospitals – free of charge.^4^ Those who enrol in this package, which supplements the public health package available to all residents, also benefit from a free annual risk assessment and prescriptions to cover up to eight weeks of treatment. Up to 2500 enrollees may be enrolled by each team. Most enrollees in Shanghai are elderly individuals with chronic diseases who have health insurance. Teams in Shanghai receive capitation payments equivalent to about US$ 20 per enrollee per year, in addition to fixed salaries. Although 60% of the capitation payment for a team is given upfront, at the start of each year, the remainder is given at the end of the year and only if the team has met set criteria on the quantity and quality of care and patient satisfaction.^25^ In 2012, capitation payments raised the income of general practitioners by 25% without significantly increasing the out-of-pocket expenditure of outpatients.^26^ The strategies followed in Shanghai’s health system encourage general practitioners to provide care of good quality and, by promoting long-term relationships between patients and their care providers,^8^ improve the continuity and comprehensiveness of the primary care that is available. In addition, Shanghai’s community health centres and hospitals are linked by an e-health system that should provide good levels of information coordination.

In Shenzhen, residents are not offered any free health services other than those available in the public health package. General practitioners in Shenzhen’s community health centres receive a fixed salary and a bonus linked with performance but no capitation payments. In Shenzhen, a fixed visit fee was implemented in all community health centres in 2012. Patients with health insurance only have to pay 30% of this fee but other patients – including about half of the megacity’s migrants –have to pay the entire fee out-of-pocket.^9^^,^^27^

In both Shanghai and Shenzhen, scores for the coordination of services fell between the first and second survey rounds – perhaps indicating a general lack of effective collaboration between community health centres and hospitals.

Shanghai’s municipal government has fully funded its community health centres, via performance-based payments, since 2006. This has improved the capacity of the human resources and preventive services in the centres.^28^ Shenzhen’s community health centres are subsidiaries of public hospitals and only marginally funded by the municipal government. Although the general practitioners working in the centres are considered to be hospital employees, they receive lower salaries than their counterparts in the hospitals.^29^ Shenzhen’s community health centres are also less well staffed and equipped than Shanghai’s. In 2011, for example, there were 3.9 general practitioners per 10 000 population in Shanghai^30^ but only 2.7 per 10 000 population in Shenzhen. All of Shanghai’s community health centres owned their offices whereas two-thirds of Shenzhen’s had to rent offices.^9^ Compared with other publicly funded community health centres, those managed by hospitals are perceived by patients to offer a lower quality of primary care^21^ and were shown to have poorer outcomes for treatment of hypertension.^31^ In Shanghai, primary care has been found to be of higher quality – and more equitable among different income groups – than that in the Hong Kong Special Administrative Region, where public providers are managed under a hospital authority.^32^

There are several strategies that might facilitate implementation of the national policy to strengthen primary care. First, the capacity of general practitioners needs to be strengthened by substantial public investment. Second, the use of capitation payments – which can encourage general practitioners to become health managers and gatekeepers^33^ – needs to be expanded. Third, to make enrolment more attractive to patients, the services provided by teams need to be tailored to the local health priorities – e.g. chronic disease among the elderly in Shanghai and maternal and child health in Shenzhen. Fourth, abolishing the fixed visit fee in Shenzhen would improve access to primary care for the megacity’s many uninsured migrants.

Countries with effective and efficient systems for primary care can achieve relatively good health outcomes at relatively low costs.^33^ Policies that help to form long-term relationships between patients and general practitioners can improve the quality of primary care. Continuity of care – which promotes mutual understanding and trust between patients and care providers – appears to be a key attribute of a successful system for primary care.
