# Corrigendum

**DOI:** 10.2471/BLT.15.100815

**Published:** 2015-08-01

In Volume 93, Issue 6, June 2015, page 407, the name of the fifth author should be “Marc KC Chong.”

